# Prolonged exposure of neonatal mice to sevoflurane leads to hyper-ramification in microglia, reduced contacts between microglia and synapses, and defects in adult behavior

**DOI:** 10.3389/fneur.2023.1142739

**Published:** 2023-03-21

**Authors:** Hong Li, Bin Zhou, Ping Liao, Daqing Liao, Linghui Yang, Jing Wang, Jin Liu, Ruotian Jiang, Lingmin Chen

**Affiliations:** ^1^Department of Anesthesiology, West China Hospital, Sichuan University, Chengdu, China; ^2^Laboratory of Anesthesia and Critical Care Medicine, National-Local Joint Engineering Research Centre of Translational Medicine of Anesthesiology, West China Hospital, Sichuan University, Chengdu, China

**Keywords:** sevoflurane, microglia, hyper-ramification, neuron, microglia-synapse contact, repopulation

## Abstract

**Background:**

Prolonged exposure to general anesthetics during development is known to cause neurobehavioral abnormalities, but the cellular and molecular mechanisms involved are unclear. Microglia are the resident immune cells in the central nervous system and play essential roles in normal brain development.

**Materials and methods:**

In the study, postnatal day 7 (P7) C57BL/6 mice were randomly assigned to two groups. In the sevoflurane (SEVO), mice were exposed to 2.5% sevoflurane for 4 h. In the control group, mice were exposed to carrier gas (30% O2/70% N2) for 4 h. Fixed brain slices from P14 to P21 mice were immunolabeled for ionized calcium-binding adapter molecule 1 (IBA-1) to visualize microglia. The morphological analysis of microglia in the somatosensory cortex was performed using ImageJ and Imaris software. Serial block face scanning electron microscopy (SBF-SEM) was performed to assess the ultrastructure of the microglia and the contacts between microglia and synapse in P14 and P21 mice. The confocal imaging of brain slices was performed to assess microglia surveillance in resting and activated states in P14 and P21 mice. Behavioral tests were used to assess the effect of microglia depletion and repopulation on neurobehavioral abnormalities caused by sevoflurane exposure.

**Results:**

The prolonged exposure of neonatal mice to sevoflurane induced microglia hyper-ramification with an increase in total branch length, arborization area, and branch complexity 14  days after exposure. Prolonged neonatal sevoflurane exposure reduced contacts between microglia and synapses, without affecting the surveillance of microglia in the resting state or responding to laser-induced focal brain injury. These neonatal changes in microglia were associated with anxiety-like behaviors in adult mice. Furthermore, microglial depletion before sevoflurane exposure and subsequent repopulation in the neonatal brain mitigated anxiety-like behaviors caused by sevoflurane exposure.

**Conclusion:**

Our experiments indicate that general anesthetics may harm the developing brain, and microglia may be an essential target of general anesthetic-related developmental neurotoxicity.

## 1. Introduction

Whether receiving general anesthesia during early life will affect the development of the central nervous system is a major scientific issue in the field of anesthesia. Animal studies suggest that inhaled and intravenous general anesthetics can lead to neurobehavioral abnormalities in adulthood (1–3), including learning and memory disorders, social disabilities, and anxiety-like behaviors (4–6). Similarly, clinical studies suggest that long-term or repeated exposure to general anesthesia, but not single or short-term exposure, can lead to abnormal behaviors, learning disabilities, and memory impairment in children (7–9). Indeed, in 2016, the US Food and Drug Administration (FDA) warned that the exposure of children under 3 years or pregnant women during their third trimester to repeated or more than 3 h of anesthesia (including sevoflurane, isoflurane, desflurane, propofol, etomidate, and ketamine.) might affect the development of children’s brains.^1^ Several high-quality clinical studies about general anesthesia neurotoxicity have been published recently. A large cohort study The Mayo Anesthesia Safety in Kids Study (MASK) reported that single anesthesia exposure before 3 years does not affect children’s overall intelligence, while multiple exposures are associated with decreased processing speed and fine motor abilities (10). General Anesthesia Compared to Spinal Anesthesia (GAS), which is the first prospective, multicenter, randomized controlled trial study, announced its findings in 2016 and 2019 (11, 12). It provided strong evidence that less than 1 h of general anesthesia in early infancy does not cause neurocognitive or behavioral deficits both at 2 and 5 years of age. More high-quality clinical studies are needed to explore the effect of prolonged or multiple general anesthesia exposures on children’s brain development.

One way in which general anesthetics compromise brain development appears to involve neurons: The anesthetics induce neuronal apoptosis and abnormal polarization, reduce spine number, and compromise synapse dysfunction (13–19). With advancements in genetic, molecular, and pharmacological tools, it is increasingly recognized that general anesthetics may also harm glial cells, which are essential for forming and maintaining neural circuits (20–22). For example, it was reported that general anesthetics could alter the cytoskeleton in immature astrocytes and delay their maturation (23, 24). In our previous study, we found that exposing neonatal mice to sevoflurane compromised astrocyte morphogenesis and led to abnormal Ca^2+^ signaling in those cells, and the animals showed abnormal social behaviors in adulthood (25).

Microglia are the resident immune cells in the central nervous system. Using their highly ramified branches to screen their microenvironment for infection, trauma, neurodegeneration, or other types of damage (26, 27), microglia can become activated to phagocytose pathogens and secrete pro-or anti-inflammatory cytokines in response to brain injury (28–30). Their dysregulation may also contribute to the pathology of neurodevelopmental disorders such as autism, Rett syndrome, and fragile X syndrome (31). In addition, microglia contribute to normal brain development by regulating neurogenesis, phagocytosing weak synapses, and modulating synapse formation and maturation (32, 33). Some studies have reported that neonatal exposure to sevoflurane induces microglial activation (34, 35).

These considerations led us to ask whether general anesthetics may exert their neurobehavioral effects by altering microglia. We explored this question here by exposing neonatal mice to sevoflurane for 4 h and then examining the effects on microglial morphology and function and the contacts between microglia and synapses, as well as the effects of microglia depletion and repopulation on neurobehavior abnormalities caused by general anesthetics.

## 2. Materials and methods

### 2.1. Animals

C57BL/6 J mice (purchased from Chengdu Da Shuo Biotechnology Co. Ltd., Chengdu, Sichuan, China) and heterozygote CX3CR1-EGFP mice (gifts from Professor Xu Ji at Zhengzhou University), which express enhanced green fluorescent protein specifically in microglia, were maintained in the Animal Feeding Center of Sichuan University with *ad libitum* access to food and drinking water. Room temperature was maintained at 22°C and fluorescent lighting was on a 12-h day–night cycle. Experimental protocols were approved by the Animal Ethics Committee of the West China Hospital of Sichuan University (protocol 2018159A) and conducted according to Sichuan University guidelines for the protection of laboratory animals.

### 2.2. Mouse model of prolonged sevoflurane exposure

On postnatal day 7, C57BL/6 J littermates were randomly divided into two groups and were put into the anesthesia induction boxes. The SEVO group mice received a gas mixture of 30% O_2_ and 70% N_2_ containing 2.5% sevoflurane (H20070172, Shanghai Hengrui Pharmaceutical Co. Ltd., Shanghai, China). The control group mice received a mixture gas of 30% of O_2_ and 70% of N_2_. The gas was delivered for 4 h at 1.5 l/min under the control of an anesthesia gas monitor (IntelliVue MP60 Anesthesia, Philips). The temperature was kept at 36–37°C using a heating pad. After the 4-h exposure, both groups of mice were returned to their mothers until the recovery of righting reflex.

### 2.3. Morphology of microglia based on immunofluorescence staining

At 7 or 14 days after sevoflurane exposure, SEVO and control mice were anesthetized with 100 mg/kg of pentobarbital, and the brain tissues were obtained after heart perfusion with phosphate-buffered solution and formalin. These tissues were fixed in formalin for 24 h at 4°C, followed by dehydration for 48 h in 30% of sucrose solution, and then sliced to a thickness of 35 μm to generate slices containing somatosensory cortex by a frozen section machine (CM1860, Leica). Following published procedures (25), we rinsed the slices in phosphate-buffered solution, incubated them in 0.5% Triton X-100 for 30 min at 37°C, and then incubated them in 10% normal goat serum for 1 h at 37°C. Microglia were immunostained for 48 h at 4°C using primary rabbit antibody against IBA1 (1:500; ab48004, Abcam, Cambridge, United States), followed by secondary 488-conjugated goat anti-rabbit antibody (1:500; ab150077, Abcam, Cambridge, United States) for 2 h at room temperature. Nuclei were stained with DAPI, and slices were sealed with an anti-fade solution and coverslips.

Total branch length, arborization area, and branch complexity of microglia in the sections were analyzed using ImageJ (version 1.53 t, National Institutes of Health, United States). Microglia were reconstructed in three dimensions using Imaris (version 7.4.2, Bitplane AG, Switzerland) and then the volumes of microglial cell bodies and branches were estimated.

### 2.4. Morphology of microglia based on the serial block face-scanning electron microscopy

The mice brain tissues were processed as described earlier and were sliced into 100-μm coronal sections, and then the layers 3–5 of the primary somatosensory cortex were isolated and were postfixed in 2% paraformaldehyde for 48 h at 4°C. After washing with phosphate-buffered solution, sections were fixed with osmium-ferricyanide and aqueous osmium tetroxide and then immersed in 2% aqueous uranyl acetate for 12–24 h at 4°C. Finally, the sections were dehydrated by a series of alcohol solutions, embedded in SPI-Pon 812 resin, and trimmed for scanning electron microscopy imaging, which was performed as previous studies described to generate image series, each of which contained 90–120 tissue sections (25, 36).

The SBF-SEM images were analyzed using Reconstruct (version 1.1.0.0, John C. Fiala). Continuous 60 images in a series were selected for three dimensions reconstruction of microglia. Microglia were identified based on their dense cytoplasm, numerous large vesicles or cellular inclusions, and elongated endoplasmic reticulum. Synapses were identified based on their shapes and the presence of presynaptic vesicles and postsynaptic density (37). To quantify contacts between microglia processes and synapses, we counted all instances when microglial processes lay directly across from dendritic spines, axon terminals, or synaptic clefts.

### 2.5. Acute brain slice preparation and two-photon confocal microscopy imaging

CX3CR1-EGFP heterozygote mice were used for two-photon confocal microscopy imaging. Acute brain slices were prepared as previously described (38). In brief, acute brain slices (300 μm) were prepared by a vibratome (VT 1200S Leica) and placed in a recording tank that was continuously perfused with the oxygenated artificial cerebrospinal fluid. Microglia in the 3–5 layer of the somatosensory cortex at a depth of 50–100 μm below the surface were imaged continuously using a 40× water immersion objective and illumination with a 488-nm laser (0.8–5% intensity) on a two-photon confocal microscope (A1R + MP system, Nikon).

Thereafter, the laser intensity was set to 100%, and the imaging area was focused on an area containing several microglia. The laser was scanned over the area for 3 s during imaging at 50X optical zoom, and a bright fluorescent sphere was observed in the tissue, indicating local laser damage. Next, the laser intensity and imaging parameters were changed back to the ones described earlier, and images were collected every 5 min for 30 min to assess the monitoring function of microglia in an activated state.

The lateral drifts of the microglia position were corrected using TurboReg Plugin in ImageJ. The pixel changes between two consecutive images were used to analyze microglia process mobility in the resting state. The microglial response to injury sites was measured by microglia processes entering from the outer area into the inner area, as the previous study described (39).

### 2.6. Microglia depletion and repopulation in neonatal mice

PLX3397 (HY-1679, Medchemexpress), an inhibitor of colony-stimulating factor 1 receptor that depletes microglia in the brain, was dissolved in 100% of dimethyl sulfoxide (DMSO) to prepare a stock solution (5 mg/ml). The 10% of the stock solution was diluted, followed by 40% of PEG300, 5% of Tween-80, and 45% of saline to obtain the final solution (0.5 mg/ml), according to the manufacturer’s protocols. This solution was injected intraperitoneally at 1 mg/kg once daily into neonatal mice from P1 to P7 to deplete microglia in the brain. Control group mice were injected with an equal amount of vehicle solution (40). Mice’s body weights were monitored throughout the treatment. At P7, both groups of mice will receive sevoflurane exposure or vehicle gas as described earlier. Moreover, part of the mice in each group was euthanized and analyzed to determine rates of microglia depletion and repopulation at P7 and P12. The rest of the mice were allowed to grow to adulthood for behavioral tests.

### 2.7. Open field test

Mice were subjected to the open field test on P42, after having been placed in the test room for environmental habituation. During the test, mice were placed in the central area of the field and allowed to explore freely for 10 min. Mouse behavior was recorded using a Smart 3.0 tracking system. The apparatus was cleaned and wiped down between tests with 30% alcohol to eliminate odor interference. Time spent in the central area and total distance traveled were calculated.

### 2.8. Elevated plus maze test

The elevated plus maze test was carried out on P44 mice, and environmental habituation was as before. At the beginning of the test, mice were placed in the center area facing the open arms. Mouse behavior was recorded for 5 min using the Smart 3.0 tracking software system. The number of entries into open or closed arms and time spent in open or closed arms were calculated.

### 2.9. Y-maze test

The Y-maze test was carried out on P47 mice. The experiment consisted of two phases. During the first phase, the novel arm was closed, and mice were placed into the starting arm and allowed to move around freely for 10 min within the starting arm and the other arm. After that, the mice were returned to their cages for 1 h. During the second phase, the novel arm was opened, and the mice were placed into the starting arm, allowing them to move around freely for 5 min within the three arms. Mouse behavior was recorded using a video camera. The number of entries into each arm and time spent in each arm during the second phase were calculated.

### 2.10. Tail suspension test

Mice were subjected to the tail suspension test on P52. The last third of the mice’s tails were bound with a 20-cm tape, and the mice were suspended from a shelf such that their heads were 15 cm above the ground. Mouse behavior was recorded for 6 min, and immobility behaviors were classified as complete motionlessness, a small movement of the forelimbs but not hind limbs, or wiggling due to inertia. Time spent immobile during the last 4 min of observation was calculated.

### 2.11. Forced swimming test

Mice were subjected to the forced swimming test on P56. One day before the test, mice were placed into the water for 15 min for “pre-swimming” preparation. After 24 h, the mice were placed into a transparent cylinder with a diameter of 10 cm and a height of 25 cm containing water at 22 ± 1°C, and their behavior was recorded for 6 min using a video camera. After the test, the mice were dried and returned to their cages. Time spent immobile during the last 4 min was calculated.

### 2.12. Statistical analysis

All statistical analyses were performed by Origin 9 software. Pairwise differences in normally distributed data were assessed for significance using a two-sample Student’s *t*-test, while pairwise differences in skewed data were assessed using a Mann–Whitney test. Differences among at least three groups were assessed using one-way analysis of Variance. Differences associated with a two-tailed *p*-value of <0.05 were considered statistically significant.

## 3. Results

### 3.1. Prolonged exposure to sevoflurane leads microglia to a hyper-ramified morphology

**Figure 1:**
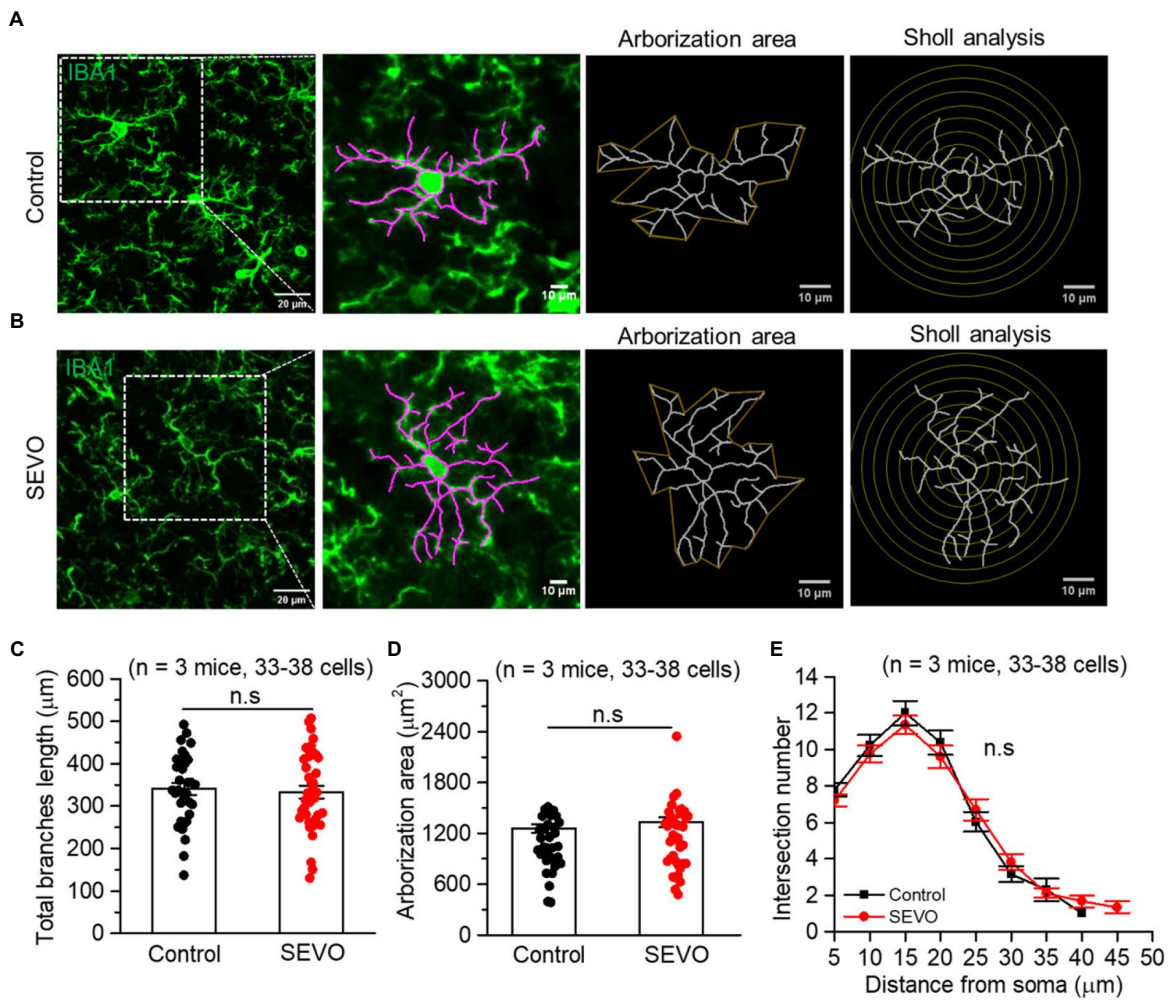
Two-dimensional morphology of microglia 7 days after prolonged neonatal sevoflurane exposure.**(A,B)** Representative images of IBA1-labeled microglia in the control group and SEVO group 7 days after prolonged neonatal sevoflurane exposure. **(C)** Quantification of the total length of microglia branches 7 days after prolonged neonatal sevoflurane exposure (*p* = 0.728, two-sample *t-*test). **(D)** Quantification of the arborization area of microglia 7 days after prolonged neonatal sevoflurane exposure (*p* = 0.313, two-sample *t-*test). **(E)** Sholl analysis of microglial morphology 7 days after prolonged neonatal sevoflurane exposure (*p* = 0.906, two-sample *t-*test). Data are shown as mean ± SEM.

**Figure 2:**
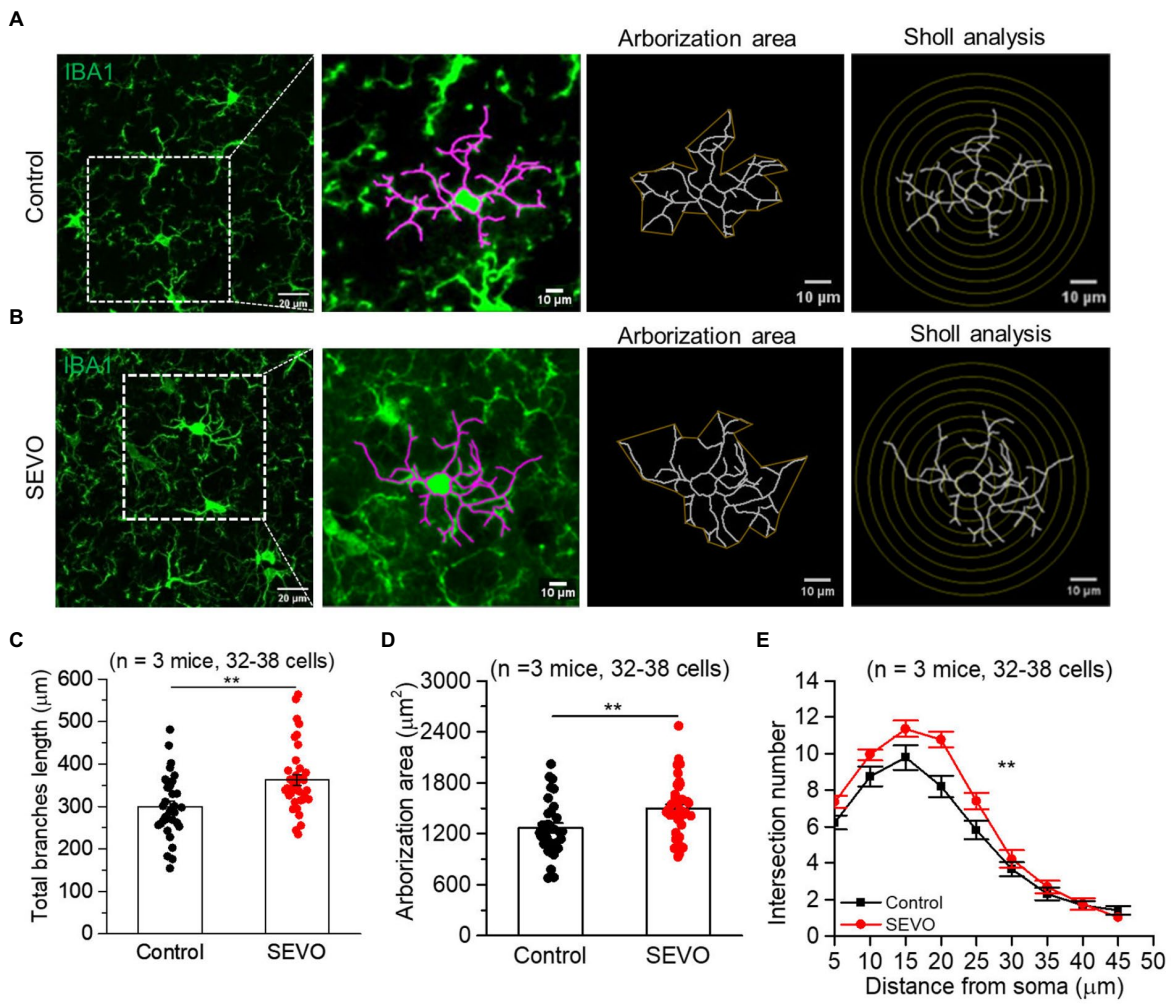
Two-dimensional morphology of microglia 14 days after prolonged neonatal sevoflurane exposure. **(A,B)** Representative images of IBA1-labeled microglia in the control group and SEVO group 14 days after prolonged neonatal sevoflurane exposure. **(C)** Quantification of the total length of microglia branches 14 days after prolonged neonatal sevoflurane exposure (*p* = 0.001, two-sample *t-*test). **(D)** Quantification of the arborization area of microglia 14 days after prolonged neonatal sevoflurane exposure (*p* = 0.005, two-sample *t-*test). **(E)** Sholl analysis of microglial morphology 14 days after prolonged neonatal sevoflurane exposure (*p* = 0.003, two-sample *t*-test). Data are shown as mean ± SEM.

**Figure 3:**
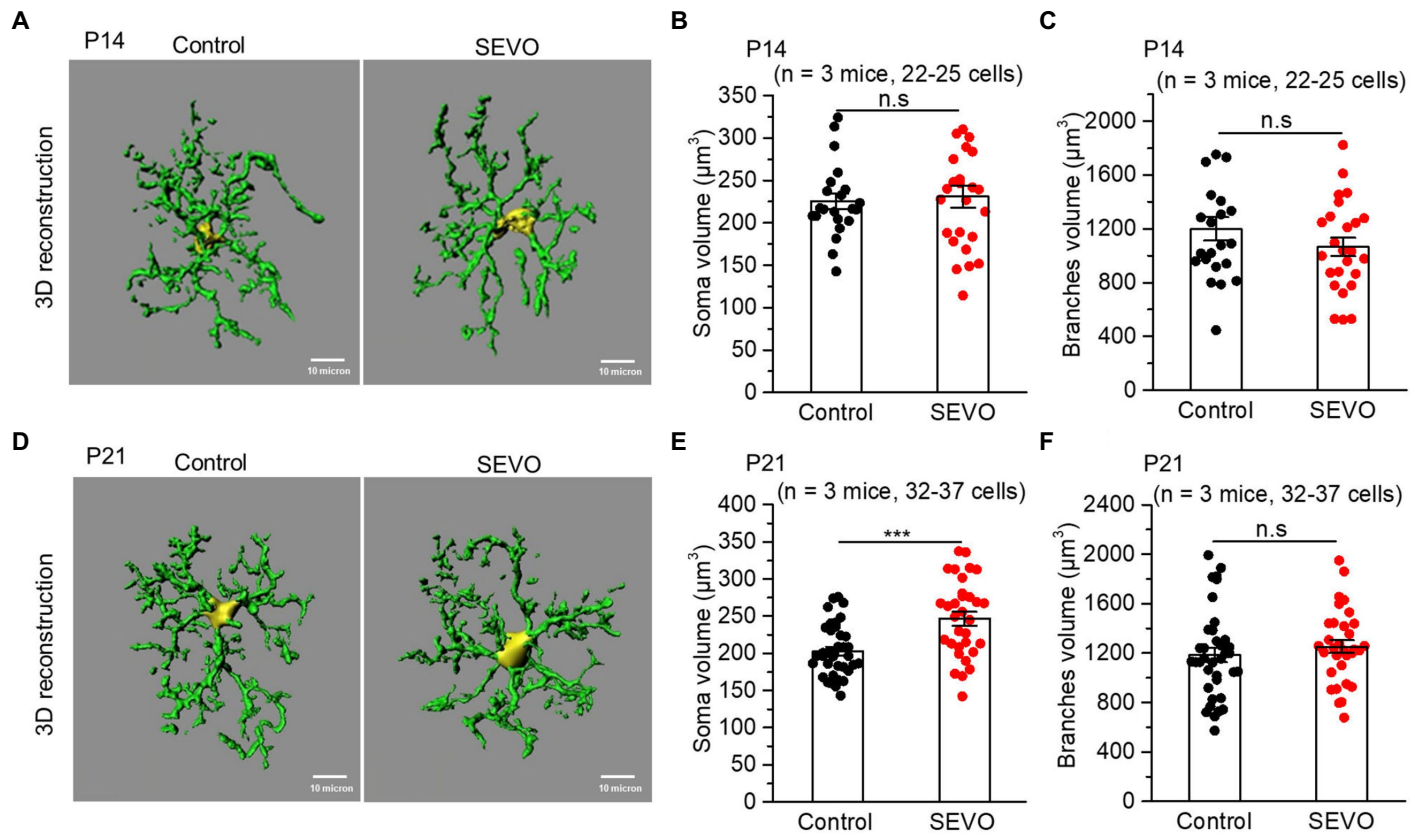
Three-dimensional morphology of microglia after prolonged neonatal sevoflurane exposure. **(A)** Representative images of IBA1-labeled microglia 3D reconstructed by Imaris software in the control and SEVO group 7 days after prolonged sevoflurane exposure. Yellow represents the cell body, and green represents branches. **(B)** Quantification of microglia soma volume 7 days after prolonged neonatal sevoflurane exposure (*p* = 0.738, two-sample *t*-test). **(C)** Quantification of microglia branches volume 7 days after prolonged neonatal sevoflurane exposure (*p* = 0.229, two-sample *t*-test). **(D)** Representative images of IBA1-labeled microglia 3D reconstructed by Imaris software in the control and SEVO group 14 days after prolonged sevoflurane exposure. Yellow represents the cell body, and green represents branches. **(E)** Quantification of microglia soma volume 14 days after prolonged neonatal sevoflurane exposure (*p* < 0.001, two-sample *t*-test). **(F)** Quantification of microglia branches volume 14 days after prolonged neonatal sevoflurane exposure (*p* = 0.399, two-sample *t*-test). Data are shown as mean ± SEM.

The prolonged exposure of neonatal mice to sevoflurane did not alter the number of Iba1 + cells or overall Iba1 fluorescence intensity (Supplementary Figure 1). We used ImageJ software to trace and evaluate the two-dimensional morphology of the microglia (Figures 1A,B and 2A,B). At 7th day after sevoflurane exposure, SEVO and control animals did not differ significantly in total branch length, arborization area, or branch complexity (Figures 1C–E). At 14th day after exposure, in contrast, SEVO animals showed 20% longer total microglial branches (Figure 2C), 18% greater microglial arborization area (Figure 2D), and greater microglial branch complexity than control animals (Figure 2E). Similarly, the two groups did not differ significantly in volumes of microglial cell bodies or branches at 7th day after sevoflurane exposure (Figures 3A–C), while SEVO group animals showed larger microglial cell volume, but not larger branch volume, than control animals at 14th day after sevoflurane exposure (Figures 3D–F). These data showed that prolonged exposure of neonatal mice to sevoflurane led to microglia morphological remodeling to a hyper-ramification state.

### 3.2. Prolonged exposure to sevoflurane does not alter the surveillance of resting or activated microglia

**Figure 4:**
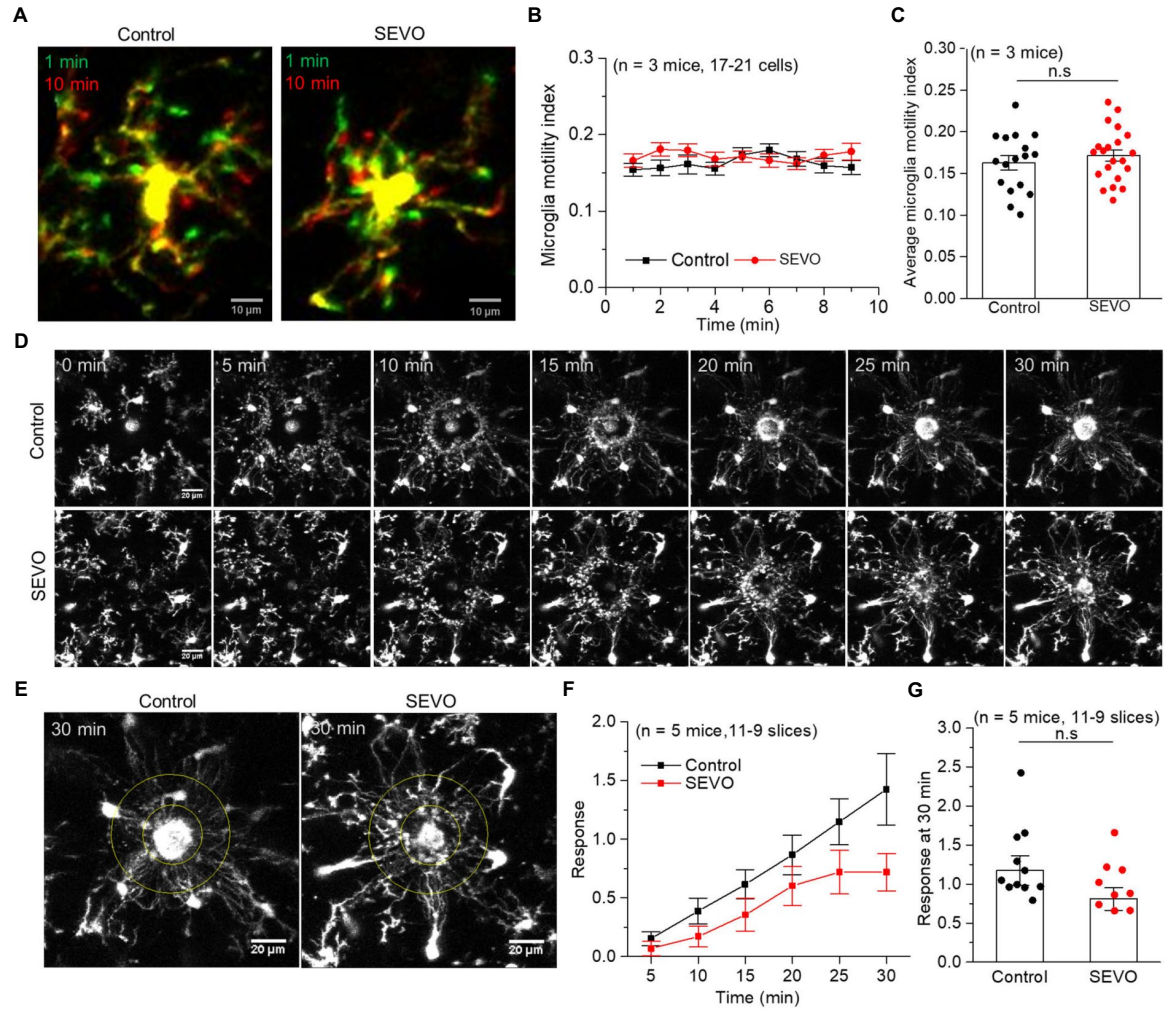
Microglia surveillance capability in resting and stimulated states 7  days after prolonged neonatal sevoflurane exposure. **(A)** Representative images of microglia in acute brain slices of CX3CR1-EGFP mice during 10-min imaging in the resting state 7 days after prolonged sevoflurane exposure. Green represents microglia at 1 min, red represents microglia at 10 min, and yellow represents the part without morphological change at 10 min compared to 1 min. **(B)** Microglia motility index analysis during 10 min imaging in the resting state. **(C)** Quantification of average microglia motility index during 10 min imaging in the resting state (*p* = 0.434, two-sample *t*-test). **(D)** Representative images of microglia response to laser burn injury within 30 min in acute brain slices of CX3CR1-EGFP mice 7 days after prolonged sevoflurane exposure. **(E)** Representative images of microglia in acute brain slices of CX3CR1-EGFP mice at 30 min after laser burn injury. **(F)** Response index of microglia within 30 min after laser burn injury of acute brain slices of CX3CR1-EGFP mice. **(G)** Quantification of response index of microglia at 30 min after laser burn injury of acute brain slices of CX3CR1-EGFP mice (*p* = 0.067, two-sample *t*-test). Data are shown as mean ± SEM.

**Figure 5:**
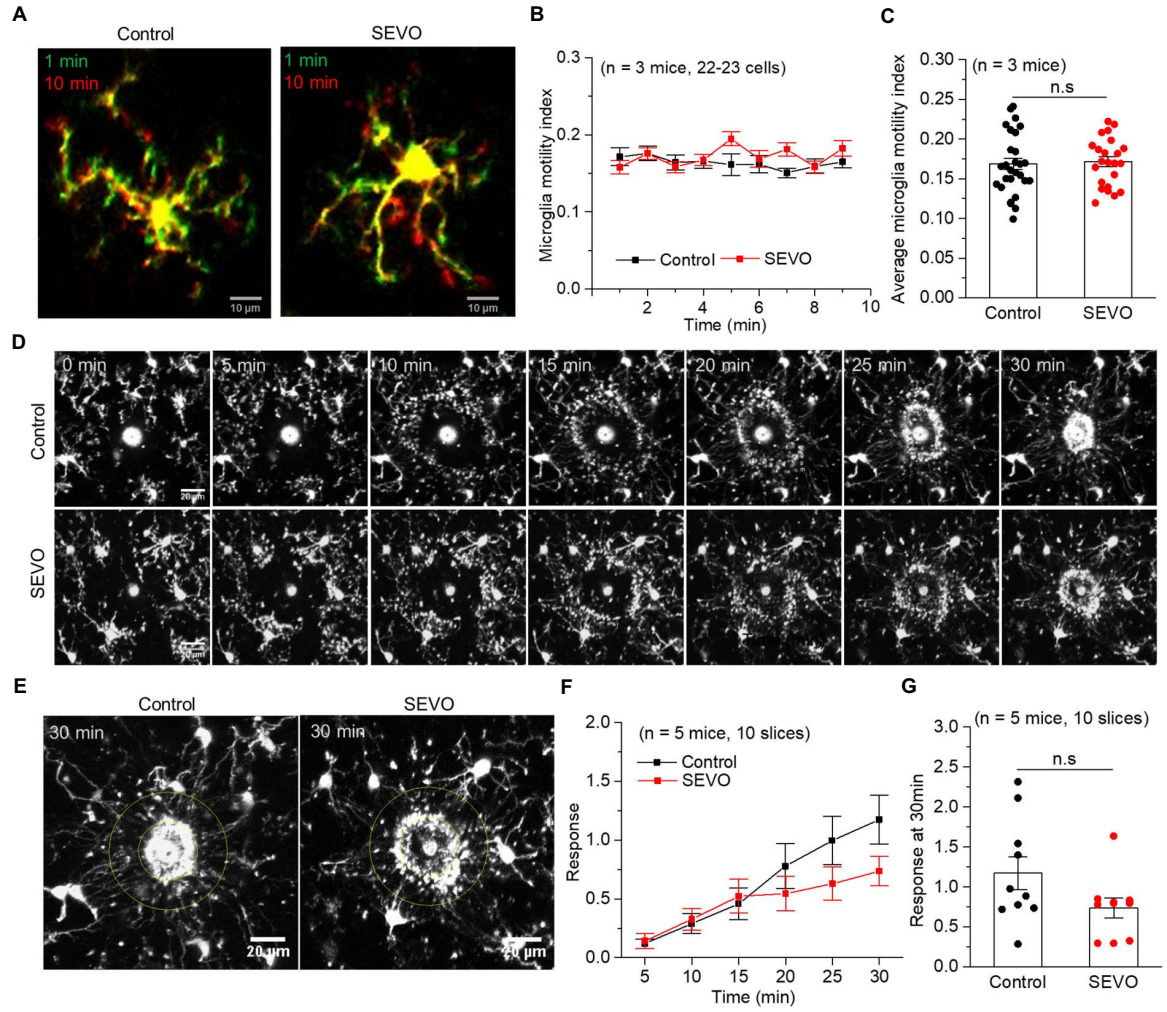
Microglia surveillance capability in resting and stimulated states 14 days after prolonged neonatal sevoflurane exposure. **(A)** Representative images of microglia in acute brain slices of CX3CR1-EGFP mice during 10-min imaging in the resting state 14 days after prolonged sevoflurane exposure. Green represents microglia at 1 min, red represents microglia at 10 min, and yellow represents the part without morphological change at 10 min compared to 1 min. **(B)** Microglia motility index analysis during 10 min imaging in the resting state. **(C)** Quantification of average microglia motility index during 10-min imaging in the resting state (*p* = 0.772, two-sample *t*-test). **(D)** Representative images of microglia response to laser burn injury within 30 min in acute brain slices of CX3CR1-EGFP mice 14 days after prolonged sevoflurane exposure. **(E)** Representative images of microglia in acute brain slices of CX3CR1-EGFP mice at 30 min after laser burn injury. **(F)** Response index of microglia within 30 min after laser burn injury of acute brain slices of CX3CR1-EGFP mice. **(G)** Quantification of response index of microglia at 30 min after laser burn injury of acute brain slices of CX3CR1-EGFP mice (*p* = 0.090, two-sample *t*-test). Data are shown as mean ± SEM.

We performed confocal imaging of EGFP-labeled microglia in acute brain slices from SEVO and control mice at 7 and 14 days after sevoflurane exposure to examine whether the sevoflurane altered the usual stretching and retraction of microglial branches as they monitored their microenvironment. We found no significant differences in the microglia motility index every minute or the average motility index during the 10 min imaging between the two groups at 7 and 14 days after exposure (Figures 4A–C and Figures 5A–C). Next, we burned the tissue locally using the laser of the confocal microscope and examined the microglial response to the injury. In both groups, microglia near the injury site extended their processes toward the damaged area, and eventually encircled the damaged site, forming a spherical containment around the injury (Figures 4D,E and Figures 5D,E). It seemed microglia branches extended slower toward the injury site in the SEVO group at 7 and 14 days after exposure, while there was no significant difference in the microglia response index between the two groups (Figures 4F,G and Figures 5F,G). Together, these data indicated that the prolonged exposure of neonatal mice to sevoflurane does not impair microglia surveillance in a resting state or their response to laser-induced damage.

### 3.3. Prolonged exposure to sevoflurane decreases contacts between microglia and synapses

**Figure 6:**
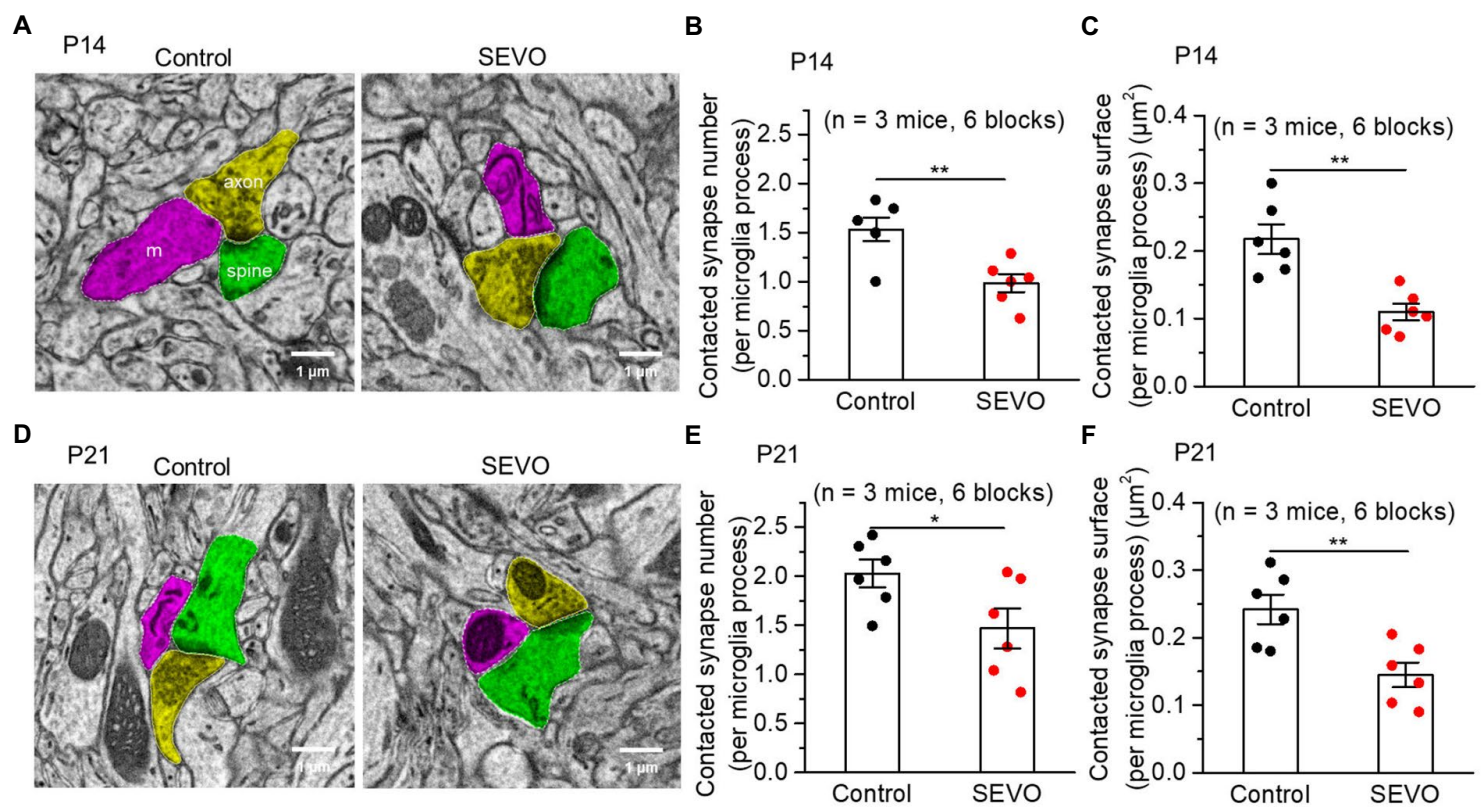
Spatial relationship between microglia and synaptic structure after prolonged neonatal sevoflurane exposure. **(A)** Representative SBF-SEM images of microglia and synapse 7 days after prolonged sevoflurane exposure. Magenta: microglia processes; Yellow: axon; Green: spine. **(B)** Quantification of synapse number contacted by per microglia process 7 days after prolonged neonatal sevoflurane exposure (*p* = 0.005, two-sample *t*-test). **(C)** Quantification of contacted synapse surface area by per microglia process 7 days after prolonged sevoflurane exposure (*p* = 0.002, two-sample *t*-test). **(D)** Representative SBF-SEM images of microglia and synapse 14 days after prolonged sevoflurane exposure. **(E)** Quantification of synapse number contacted by per microglia process 14 days after prolonged neonatal sevoflurane exposure (*p* = 0.047, two-sample *t*-test). **(F)** Quantification of contacted synapse surface area by per microglia process 14 days after prolonged sevoflurane exposure (*p* = 0.007, two-sample *t*-test). Data are shown as mean ± SEM.

Whether prolonged sevoflurane exposure will affect the contacts between microglia and synapse in neonatal mice, which is vital for forming and pruning synapses (32, 33, 41), is unclear. Using SBF-SEM (Figures 6A,D), we examined the contacts between microglia and synapses in SEVO and control mice at 7 and 14 days after sevoflurane exposure. At both time points, the two groups did not differ significantly in volumes of microglial processes (Supplementary Figure 2), but the SEVO group showed a significant decrease in microglia-synapse contact number (Figures 6B,E) and smaller total microglia-synapse contact area (Figures 6C,F). These results indicated that prolonged exposure to sevoflurane decreases microglia-synapse interactions.

### 3.4. Microglia depletion and repopulation in the neonatal brain ameliorate sevoflurane-induced anxiety-like behavior in adulthood

**Figure 7:**
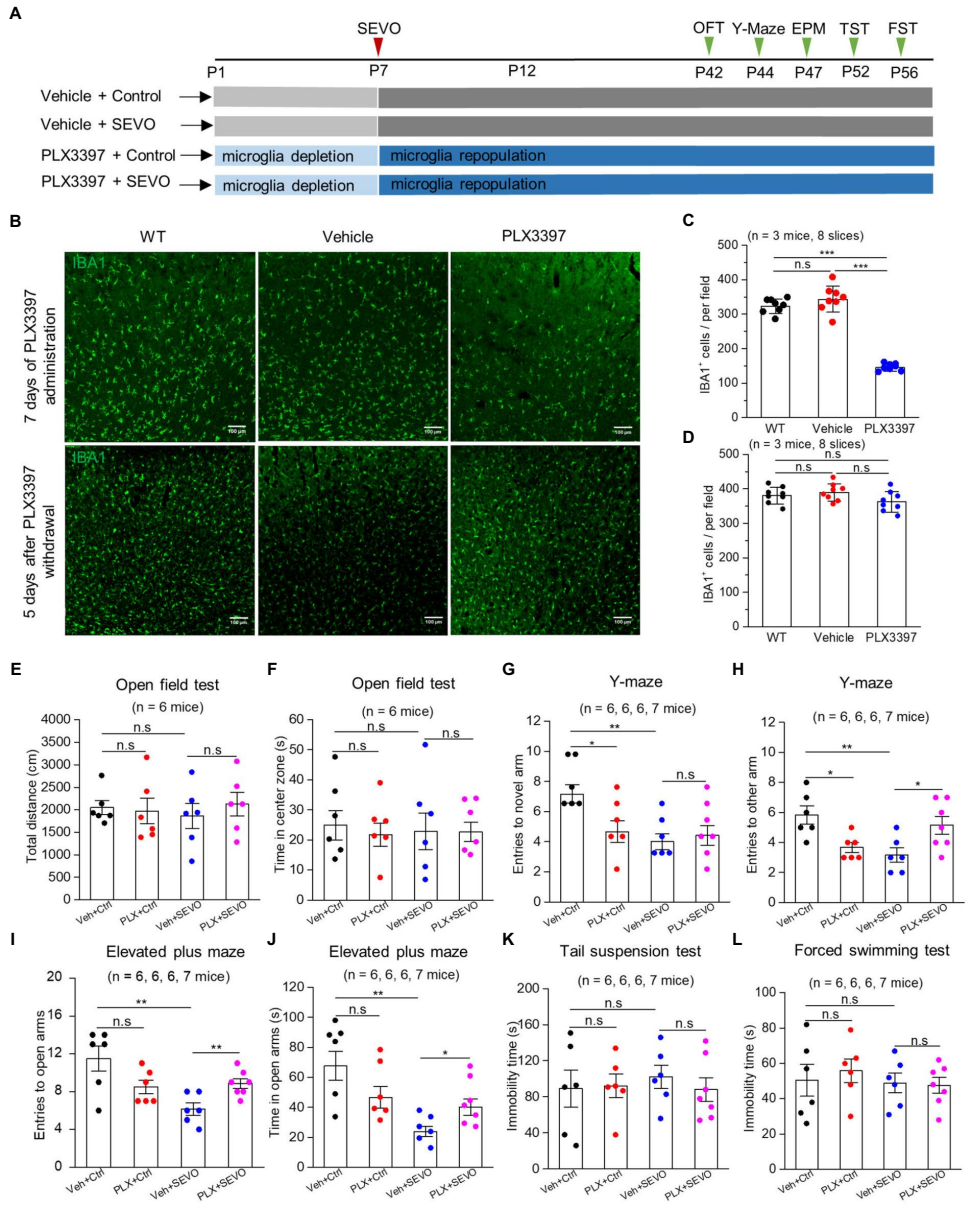
Microglia depletion before sevoflurane exposure and later neurobehavioral experiments in adult mice. **(A)** Flow chart of microglia depletion by PLX3397 before sevoflurane exposure and neurobehavioral experiments in adult mice. **(B)** Representative images of IBA1 expression in the somatosensory cortex after 7 days of PLX3397 administration (above), and 5 days after PLX3397 withdrawal (below). **(C)** Quantification of the number of IBA1^+^ cells after 7 days of PLX3397 administration (WT vs. Veh: *p* = 0.211; WT vs. PLX: *p* < 0.001; Veh vs. PLX: *p* < 0.001, one-way ANOVA test). **(D)** Quantification of the number of IBA1^+^ cells 5 days after PLX3397 withdrawal (WT vs. Veh: *p* = 0.489; WT vs. PLX: *p* = 0.204; Veh vs. PLX: *p* = 0.073, one-way ANOVA test). **(E)** Quantification of total distance in the open field test (Veh + Ctrl vs. PLX + Ctrl: *p* = 0.822; Veh + Ctrl vs. Veh + SEVO: *p* = 0.566; Veh + SEVO vs. PLX + SEVO: *p* = 0.504, one-way ANOVA test). **(F)** Quantification of time in the center zone in the open field test (Veh + Ctrl vs. PLX + Ctrl: *p* = 0.623; Veh + Ctrl vs. Veh + SEVO: *p* = 0.803; Veh + SEVO vs. PLX + SEVO: *p* = 0.971, one-way ANOVA test). **(G)** Quantification of entries to novel arm in the Y-maze test (Veh + Ctrl vs. PLX + Ctrl: *p* = 0.023; Veh + Ctrl vs. Veh + SEVO: *p* = 0.001; Veh + SEVO vs. PLX + SEVO: *p* = 0.852, one-way ANOVA test). **(H)** Quantification of entries to the other arm in the Y-maze test (Veh + Ctrl vs. PLX + Ctrl: *p* = 0.010; Veh + Ctrl vs. Veh + SEVO: *p* = 0.006; Veh + SEVO vs. PLX + SEVO: *p* = 0.028, one-way ANOVA test). **(I)** Quantification of entries to open arm in the elevated plus test (Veh + Ctrl vs. PLX + Ctrl: *p* = 0.076; Veh + Ctrl vs. Veh + SEVO: *p* = 0.005; Veh + SEVO vs. PLX + SEVO: *p* = 0.007, one-way ANOVA test). **(J)** Quantification of time in open arm in the elevated plus test (Veh + Ctrl vs. PLX + Ctrl: *p* = 0.113; Veh + Ctrl vs. Veh + SEVO: p = 0.002; Veh + SEVO vs. PLX + SEVO: *p* = 0.031, one-way ANOVA test). **(K)** Quantification of immobility time in tail suspension test (Veh + Ctrl vs. PLX + Ctrl: *p* = 0.899; Veh + Ctrl vs. Veh + SEVO: *p* = 0.608; Veh + SEVO vs. PLX + SEVO: *p* = 0.470, one-way ANOVA test). **(L)** Quantification of immobility time in forced swimming test (Veh + Ctrl vs. PLX + Ctrl: *p* = 0.633; Veh + Ctrl vs. Veh + SEVO: *p* = 0.890; Veh + SEVO vs. PLX + SEVO: *p* = 0.861, one-way ANOVA test). Data are shown as mean ± SEM.

We repeated the sevoflurane exposure with neonatal mice after pretreating them with CSF-1R inhibitor PLX3397 (Figure 7A), which eliminated approximately 60% of microglia from the brain at P7, and microglia repopulated completely 5 days after PLX3397 withdrawal (Figures 7B–D). Microglia depletion neither affected body weight or locomotion during young adulthood (Supplementary Figure 3 and Figures 7E,F) nor induced anxiety-or depression-like behaviors (Figures 7I–L). However, microglia depletion decreased entries into the novel arm and other arms in the Y-maze test in adulthood (Figures 7G,H), indicating spatial memory impairment. Neonatal prolonged sevoflurane did not affect the locomotive ability or cause depression-like behaviors (Figures 7E,F,K,L), but it did disrupt spatial memory in the Y-maze test and led to anxiety-like behavior in the elevated plus maze (Figures 7G–J). Microglia depletion before sevoflurane exposure and repopulation did not ameliorate the spatial memory deficit caused by neonatal sevoflurane exposure (Figures 7G,H), but it did ameliorate anxiety-like behavior induced by neonatal sevoflurane exposure (Figures 7I,J).

## 4. Discussion

In this study, we confirm and extend previous reports implicating microglial alterations in the adverse neurobehavioral effects of prolonged neonatal exposure to general anesthesia (34, 35). We provide evidence in a mouse model that prolonged neonatal sevoflurane exposure induces hyper-ramification of microglia, reduces contacts between microglia and synapse, and leads to subsequent anxiety-like behavior in adulthood. We also show evidence that microglia depletion and repopulation in the neonatal brain can mitigate sevoflurane-induced anxiety-like behavior in adulthood.

In our mouse model, neonatal sevoflurane exposure did not lead to the microglial activation reported in other studies involving multiple neonatal sevoflurane exposures (34, 35). Instead, exposure in our mouse model led to microglia hyper-ramification with an increase in total branch length, coverage area, and branch complexity at 14 days post-exposure. Such hyper-ramification morphological changes have been observed in animal models of post-traumatic stress disorder, chronic despair, and aging (42–44). These morphological changes indicate an intermediate stage between resting microglia and reactive microglia, suggesting that our model of prolonged sevoflurane exposure-induced relatively mild damage to the developing brain. Consistently, Liu Yu et al. found that acute isoflurane exposure led to the hyper-ramification of microglia in awake mice (45). Little was known about the mechanism to initiate microglia hyper-ramification. Intact CX3CL1-CX3CR1 signaling was acquired for microglia hyper-ramification in the chronic despair model (43). The mechanism of neonatal sevoflurane exposure-induced microglia hyper-ramification remains to be established.

Microglia continually extend and retract their branches in all directions under physiological conditions, allowing them to monitor the brain microenvironment (46). In response to injury, microglia become activated and extend their branches toward the injury site. We found that prolonged neonatal exposure to sevoflurane neither affected the surveillance of resting microglia nor significantly reduced their response to the laser-induced injury site. In contrast, Madry C et al. found that isoflurane or sevoflurane exposure to acute brain slices inhibited microglia surveillance in the resting state (47). While Sun et al. reported that microglia branches extended more rapidly to the laser-induced injury after acute exposure to sevoflurane (48). These different results above indicate that acute anesthetics’ interference with microglia surveillance may not have long-lasting effects.

We found that prolonged neonatal exposure to sevoflurane reduced contacts between microglia and synapse. Such contacts, which depend on neuronal activity (49, 50), can lead to new synapse formation (33), as well as the pruning of the existing synapse (37). Our results provide evidence that reduced microglia-synapse contacts due to neonatal sevoflurane exposure may link altered neuronal signaling to behavioral defects in adulthood.

CSF-1R inhibitor-induced microglial depletion and repopulation after withdrawal did not induce inflammation, cytokine storm, or blood–brain barrier damage and had no apparent adverse effects on general animal health and neurobehaviors (51, 52). It was reported that the repopulated microglia were generated from the original residual microglia after microglia clearance (53), and the repopulating microglia resemble normal ramified microglia by 14 days after PLX3397 withdrawal (51). In our mouse model, the partial depletion of microglia from the neonatal brain before prolonged exposure to sevoflurane and later microglia repopulation can ameliorate sevoflurane-induced anxiety-like behavior in adulthood. Microglial depletion and repopulation have also shown impressive results in some animal models of neurological diseases and deficits, including traumatic brain injury, maternal immune activation-induced repetitive behavior, social deficits, and Parkinson’s disease (54–57). These results support continuing research into the potential of microglial depletion and repopulation for treating neurological disorders and deficits.

## Data availability statement

The raw data supporting the conclusions of this article will be made available by the authors, without undue reservation.

## Ethics statement

The animal study was reviewed and approved by the Animal Ethics Committee of the West China Hospital of Sichuan University.

## Author contributions

RJ and LC conceived and designed the study. HL, LC, and BZ conducted the experiments and collected the data. HL, LC, PL, and DL conducted the immunofluorescence staining and confocal microscopy imaging. LC, HL, JW, and LY conducted animal behavior tests. LC and HL analyzed the data and wrote the original draft of the manuscript. LC, HL, and RJ reviewed and edited the manuscript. RJ and JL supervised the study. All authors contributed to the article and approved the submitted version.

## Funding

This study was supported by the Department of Science and Technology of Sichuan Province, China (No: 2020YFS0186) and the National Natural Science Foundation of China (No: 82001130).

## Conflict of interest

The authors declare that the research was conducted in the absence of any commercial or financial relationships that could be construed as a potential conflict of interest.

## Publisher’s note

All claims expressed in this article are solely those of the authors and do not necessarily represent those of their affiliated organizations, or those of the publisher, the editors and the reviewers. Any product that may be evaluated in this article, or claim that may be made by its manufacturer, is not guaranteed or endorsed by the publisher.

## Footnotes

^1^ https://www.fda.gov/drugs/drugsafety/ucm532356.htm
