# Supplementary material for: Prolonged exposure of neonatal mice to sevoflurane leads to hyper-ramification in microglia, reduced contacts between microglia and synapses, and defects in adult behavior
Source: Front Neurol. 2023 Mar 21;14:1142739. doi: 10.3389/fneur.2023.1142739 (PMC10072331; doi:10.3389/fneur.2023.1142739)
Supplement: Supplementary file 1 [file Table_1.DOCX]

***Supplementary Material***

**Prolonged exposure of neonatal mice to sevoflurane leads to hyper-ramification in microglia, reduced contacts between microglia and** **synapses, and defects in adult behavior**

**Hong Li, Bin Zhou, Ping Liao, Daqing Liao, Linghui Yang, Jing Wang，Jin Liu, Ruotian Jiang*, Lingmin Chen***

*** Correspondence:**

Ruotian Jiang*; Lingmin Chen*

ruotianjiang@scu.edu.cn; 1010702289@qq.com

1. **Supplementary Figures**


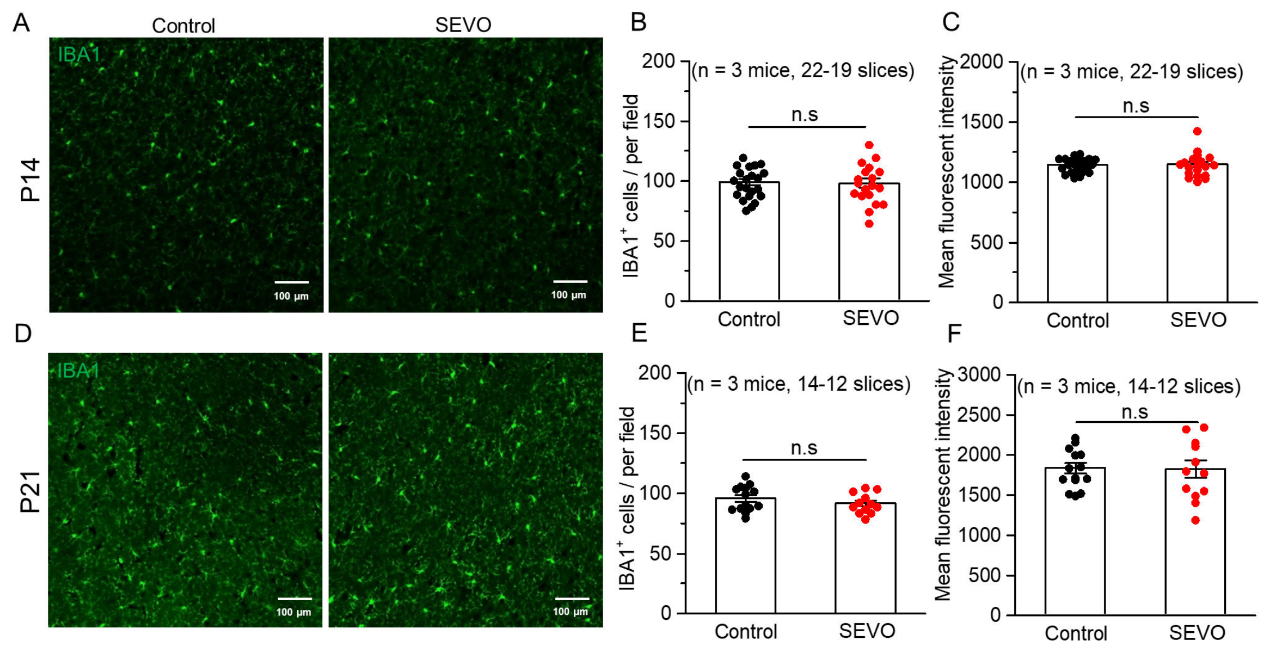


**Supplemental Fig. 1 IBA1 expression in somatosensory cortex after prolonged neonatal sevoflurane exposure**

(A) Representative images of IBA1 expression in somatosensory cortex 7 days after prolonged neonatal sevoflurane exposure. (B) Quantification of IBA1^+^ cell number 7 days after prolonged neonatal sevoflurane exposure (*P* = 0.864, two sample *t* test). (C) Quantification of IBA1 fluorescent intensity 7 days after prolonged neonatal sevoflurane exposure (*P* = 0.990, two sample *t* test). (D) Representative images of IBA1 expression in somatosensory cortex 14 days after prolonged neonatal sevoflurane exposure. (E) Quantification of IBA1^+^ cell number 14 days after prolonged neonatal sevoflurane exposure (*P* = 0.281, two sample *t* test). (F) Quantification of IBA1 fluorescent intensity 14 days after prolonged neonatal sevoflurane exposure (*P* = 0.898, two sample *t* test). Data are shown as mean ± SEM.


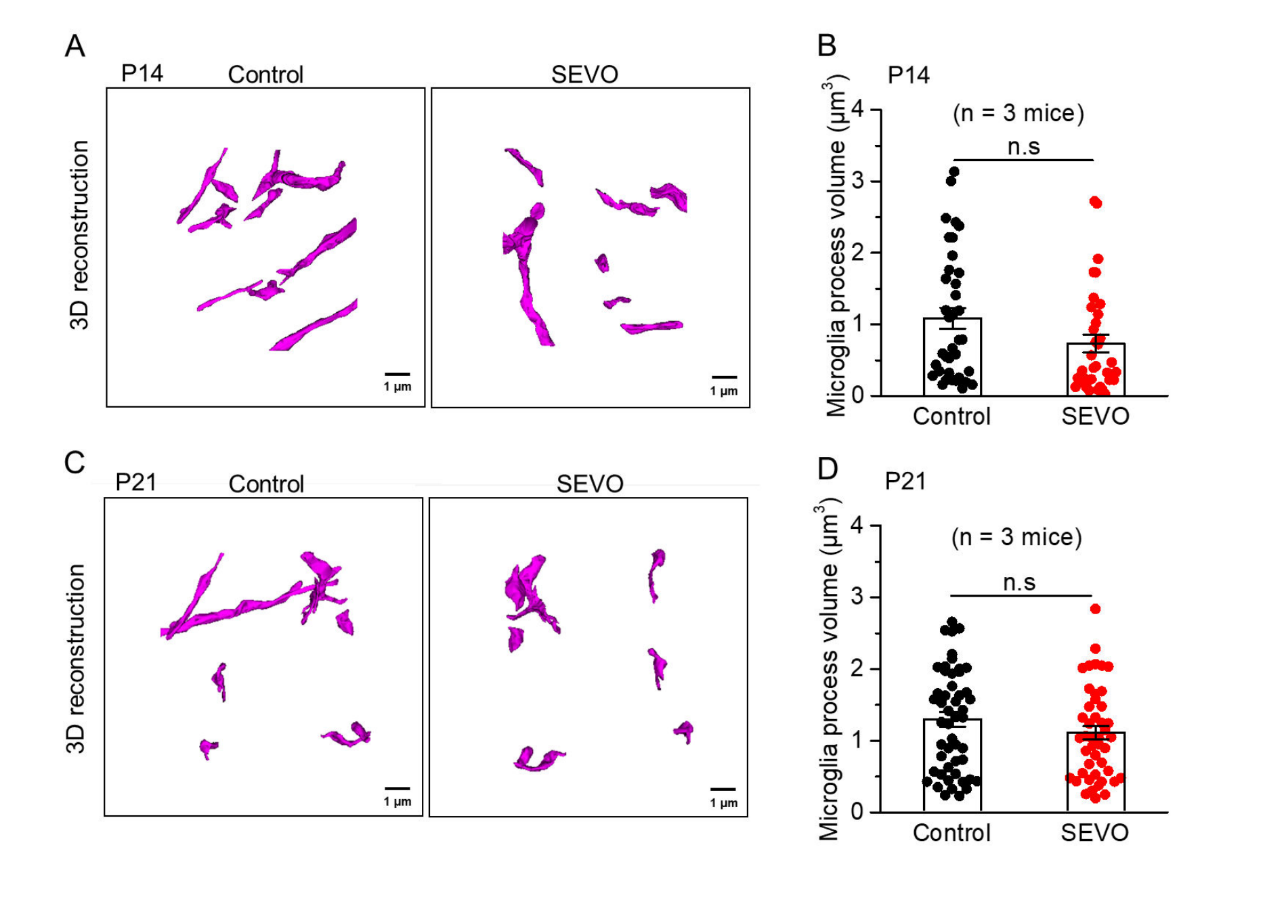


**Supplemental Fig. 2 Microglia process volume after prolonged sevoflurane exposure.**

(A) Representative images of 3D reconstruction microglia process in scanning electron microscope 7 days after prolonged neonatal sevoflurane exposure. (B) Quantification of single microglia process volume (*P* = 0.072, two sample *t* test). (C) Representative images of 3D reconstruction microglia process in scanning electron microscope 14 days after prolonged neonatal sevoflurane exposure. (D) Quantification of single microglia process volume (*P* = 0.185, two sample *t* test). Data are shown as mean ± SEM.


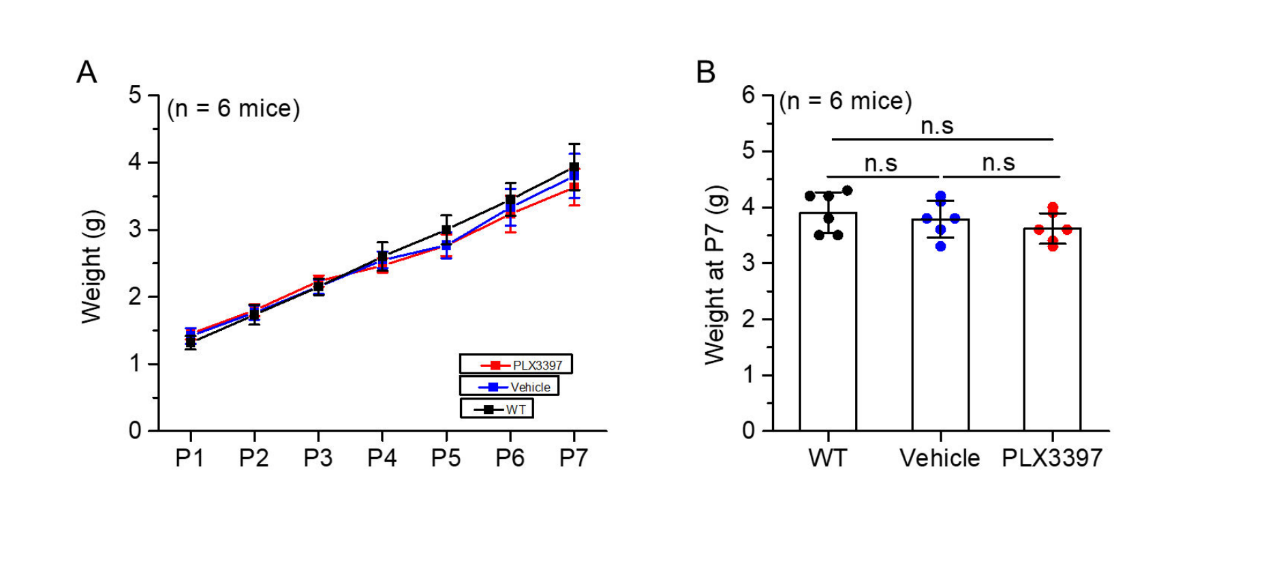


**Supplemental Fig. 3 Mice weight during PLX3397 treatment.**

(A) Mice weight from P1 to P7 in WT, vehicle and PLX3397 treatment group. (B) Mice weight at P7 in three groups. (WT *vs* Veh: *P* = 0.574; WT *vs* PLX3397: *P* = 0.159; Veh *vs* PLX: *P* = 0.362, one-way ANOVA test). Data are shown as mean ± SEM.
